# Phorbol esters in seed oil of *Jatropha curcas* L. (saboodam in Thai) and their association with cancer prevention: from the initial investigation to the present topics

**DOI:** 10.1007/s00432-017-2341-6

**Published:** 2017-01-25

**Authors:** Hirota Fujiki, Maitree Suttajit, Anchalee Rawangkan, Keisuke Iida, Pornngarm Limtrakul, Sonthaya Umsumarng, Masami Suganuma

**Affiliations:** 1 Faculty of Medicine, Saga University, Nabeshima, Saga, 849-8501 Japan; 2 School of Medical Sciences, University of Phayao, Phayao, 56000 Thailand; 3 Graduate School of Science and Engineering, Saitama University, Sakura-ku, Saitama, 338-8570 Japan; 4 Department of Biochemistry, Faculty of Medicine, Chiang Mai University, Chiang Mai, 50200 Thailand

**Keywords:** DHPB, Economical fuel, *Jatropha curcas* L., Seed oil, Tumor promoter

## Abstract

**Purpose:**

In 1988, we first reported the complete chemical structure of a new type of phorbol ester, abbreviated to DHPB, found in seed oil of *Jatropha curcas* L. (Saboodam in Thai) and its tumor-promoting activity on mouse skin. Although this seed oil contains toxic phorbol ester, it was planned to use it as a feasible renewable oil and the extracted seed cake as fertilizer. This utilization value opened a new science of *Jatropha curcas*.

**Methods:**

The main experimental results are cited from our publications, and the relevant literature screened from journals and PubMed.

**Results and discussion:**

This paper begins with our original work on the structural elucidation of a new phorbol ester, 12-deoxy-16-hydroxyphorbol (DHPB): its tumor-promoting activity was compared with that of TPA. We think that it is timely to review the following research advances with *Jatropha curcas*, so numerous topics are classified as follows: (1) historical development of phorbol esters in seed oil; (2) toxicity of phorbol ester based on various bioassays; (3) degradation of phorbol ester; (4) a new pharmaceutical compound in seed; and (5) tumor promotion and progression with endogeneous tumor promoters in human carcinogenesis. The discovery of phorbol ester in seed oil raised awareness of the danger of public use of seed oil and seed cake in Thailand, and also indicated the necessity of discussing the concept of primary and tertiary cancer preventions.

**Conclusion:**

It is worthwhile to study the future benefits and cancer risks of globally distributed *Jatropha curcas* L.

## Introduction

**Fig. 1:**
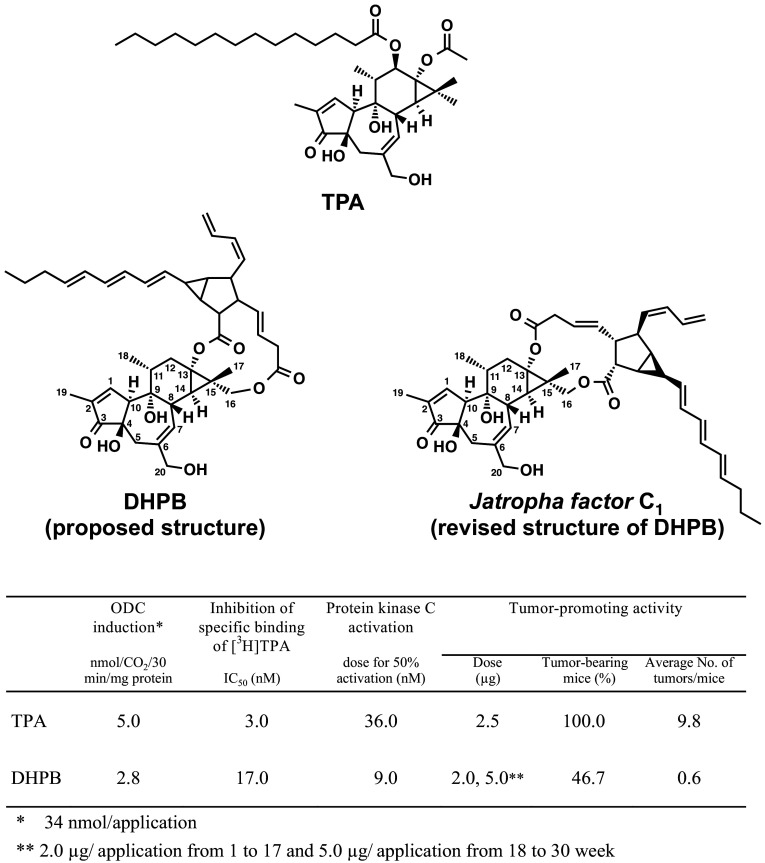
Structures of the classic tumor promoter, 12-*O*-tetradecanoylphorbol-13-acetate (TPA) in the center, 12-deoxy-16-hydroxyphorbol-4′-[12′,14′-butadienyl]-6′-[16′,18′,20′-nonatrienyl]-bicyclo[3.1.0]hexane-(13-*O*)-2′-[carboxylate]-(16-*O*)-3′-[8′-butenoic-10′]ate (abbreviated to DHPB), and proposed structure on the left and revised structure on the right (named *Jatropha factor* C_1_), along with their biochemical and biological activities

*Jatropha curcas* L. (Saboodam in Thai language) is commonly grown around houses, as a fence to keep out cattle and other animals in Asian countries and Africa. The Saboodam is a shrub about 2–5 m high. The original meaning of Saboodam is black soap, and its seed oil for a long time used as a material for making soap, lamp oil, and candles. Moreover, seed oil is now used in dye-paint and printing ink. During the oil crisis in the 1970s, seed oil attracted great attention as an alternative fuel (renewable oil) for water pumps, motor cycles, and other engines. Therefore, the cultivation of Saboodam was strongly promoted by Thai agricultural authorities and other nations (Suttajit et al. 1990). *Jatropha curcas* L. belongs to the family *Euphorbiaceae*, which includes *Croton tiglium* L., which contains a classic tumor promoter, 12-*O*-tetradecanoylphorbol-13-acetate (TPA, Fig. 1) (Hecker et al. 1967). In 1984, Erich Hecker and his associates at German Cancer Research Center reported that seed oil of *Jatropha* species contained polyunsaturated esters of 12-deoxy-16-hydroxyphorbol, as an irritant (Adolf et al. 1984). Although Hecker is a pioneer in the structural elucidation of TPA from croton oil, he did not complete the structural determination of the irritant, since polyunsaturated acid moieties are extremely unstable. If seed oil of *Jatropha curcas* contains an irritant that has tumor-promoting activity on mouse skin, we are concerned that the wide utilization and exposure of seed oil will be a health risk to a large population in Thailand. From the standpoint of primary cancer prevention, we decided to elucidate the complete chemical structure of the irritant in oil of *Jatropha curcas*, and determine its tumor-promoting activity on mouse skin. We did this in a two-stage carcinogenesis experiment.

In 1984, an Overseas Scientific Research Survey (Cancer Program) from the Ministry of Education, Science and Culture, Japan, encouraged us to challenge this scientific project, based on strong collaboration with Vichai Wongchai and Maitree Suttajit at the Department of Biochemistry, Faculty of Medicine, Chiang Mai University, Thailand (Horiuchi et al. 1987). Next, we showed that the irritant in seed oil contained a new type of phorbol ester, an intramolecular 13,16-diester of 12-deoxy-16-hydroxyphorbol (abbreviated to DHPB, Fig. 1), and found that DHPB has a weaker tumor-promoting activity than TPA, in a two-stage carcinogenesis experiment on mouse skin initiated with 7,12-dimethylbenz(a)anthracene (DMBA) (Hirota et al. 1988). The results were reported by Hirota Fujiki’s research group at the National Cancer Center Research Institute in Tokyo, Koichi Shudo at University of Tokyo, and Suttajit and Wongchai in Thailand, along with Hecker in Germany (Hirota et al. 1988). The finding of an active tumor promoter in seed oil taught Thai farmers to take care to avoid direct contact with seed oil of *Jatropha curcas* during oil extraction (Fujiki et al. 1987; Suttajit et al. 1990). Immediately, Thai scientists, such as Suttajit and Wongchai, announced the possible hazard of Saboodam seed oil in a large number of Thai publications, books, and journals, and emphasized the concept of primary cancer prevention (Fujiki et al. 2012).

Since we published the discovery of DHPB and its tumor-promoting activity in 1988, numerous investigators have reported new results in *Jatropha curcas* research. We think that it is time to briefly review recent results with *Jatropha curcas*, and the topics of this review are as follows: (1) phorbol ester as a tumor promoter and its tumor promotion; (2) a new phorbol ester (DHBP) in seed oil and its tumor-promoting activity on mouse skin; (3) the toxicity of phorbol ester in seed oil and seed cake as determined by various bioassays; (4) degradation of phorbol ester in utilization of seed oil and seed cakes; (5) new pharmaceutical compound isolated from seed of *Jatropha curcas*; (6) tumor promotion and progression with endogeneous tumor promoters in human carcinogenesis; and (7) concept of primary and tertiary cancer preventions. In brief, *Jatropha curcas*, Saboodam, provides important research sources.

## Phorbol ester as a tumor promoter and its tumor promotion

**Fig. 2:**
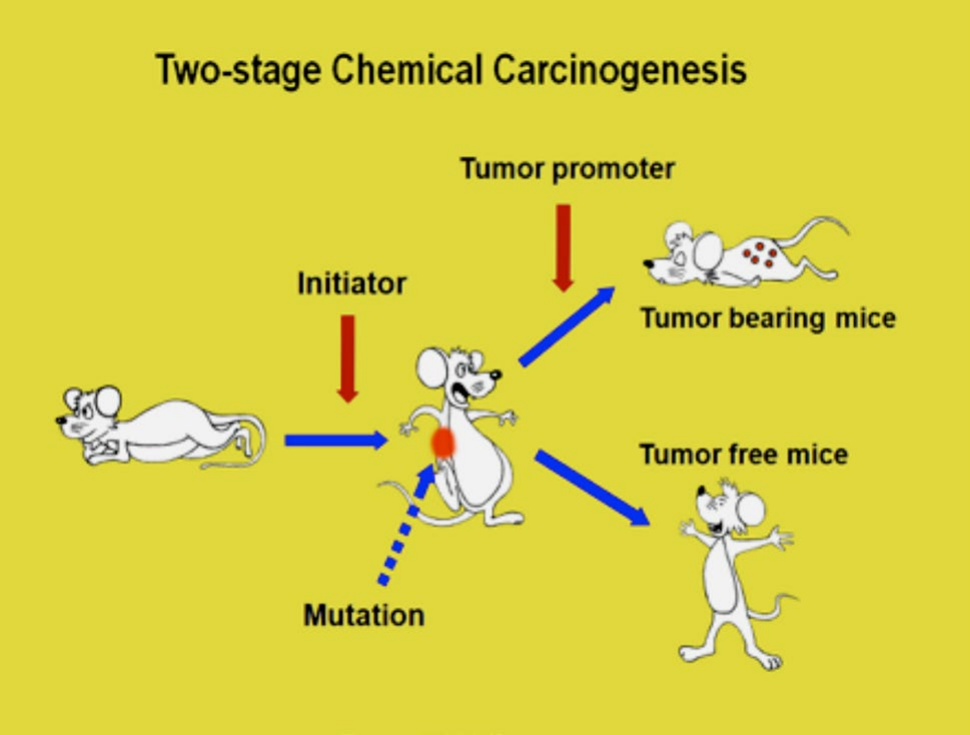
Two-stage chemical carcinogenesis on mouse skin, initiation, and tumor promotion. Initiation is a single application of a small amount of carcinogen, called an initiator, and tumor promotion is clonal growth of initiated cells by repeated applications of tumor promoter

*Jatropha curcas* L. and *Croton tiglium* L. belong to *Euphorbiaceae*. Croton oil of *Croton tiglium* has strong tumor-promoting activity based on classic two-stage chemical carcinogenesis experiments on mouse skin model, established in the 1940s by Issac Berenblum (1941) and J. C. Mottram (1944) (Fig. 2). 12-*O*-Tetradecanoylphorbol-13-acetate (TPA) was isolated from croton oil and chemically elucidated by Hecker and his associates as the most potent tumor promoter (Fig. 1) (1967). Thus, seed oil of *Jatropha curcas* Saboodam is thought to contain a tumor promoter of the phorbol ester class. Theoretically, two-stage chemical carcinogenesis, consisting of initiation and tumor promotion, is now understood as follows (Fig. 2). Experimentally, a single application of 100 μg of 7,12-dimethylbenz(a)anthracene (DMBA) induces irreversible mutation in mouse skin, and DMBA is named an initiator. 1 μg of TPA is repeatedly applied to mouse skin previously treated with DMBA produces high percentages of tumor-bearing mice, usually 90–100%. The biological activity of TPA is called tumor-promoting activity, and TPA is now called a tumor promoter. A single application of DMBA alone or repeated applications of TPA alone do not produce any tumors: a tumor promoter specifically induces clonal growth of the initiated cells, resulting in tumor development (Fujiki et al. 1981).

During our investigation of tumor promoters, we used the irritant activity on mouse skin as a biomarker, and identified numerous tumor promoters, such as teleocidin A (A-1 and A-2) and B (B-1~B-4) from *Streptomyces mediocidicus*, lyngbyatoxin A (teleocidin A-1), aplysiatoxin, and debromoaplysiatoxin from blue–green algae (Fujiki and Sugimura 1987). Although the chemical structures of the tumor promoters are different from those of TPA and DHPB, they both bound to the phorbol ester receptor in cell membrane and activated protein kinase C (Fujiki et al. 1984a). Because they both induced tumor promotion through the same mechanisms of action, we called these tumor promoters the TPA-type tumor promoters (Fujiki et al. 1984b). All the results indicated that numerous potent tumor promoters, with various chemical structures, are present in our environment. The study taught us the need to avoid direct contact with tumor promoters in our every-day life, which is called primary cancer prevention.

## A phorbol ester (DHBP) in seed oil and its tumor-promoting activity on mouse skin

The seed and seed oil of *Jatropha curcas* were obtained from the Division of Agricultural Chemistry, Department of Agriculture, Bangkok Thailand. Using irritant activity on mouse ear as a biomarker, Takahiko Horiuchi and his associates partially purified “irritant fraction I” from the methanol extract of seed oil by column chromatographies on Florisil and Sephadex LH-20. Further purification of “irritant fraction I” by LiChroprep RP18 column with 85% acetonitrile resulted in “irritant fraction II”. Application of 5 μg of “irritant fraction I” showed a ++ grade of irritation and that of “irritant fraction II” gave a +++ grade, while a + grade of irritation was induced by 3.4 ng of teleocidin, a TPA-type tumor promoter. Since “irritant fractions I and II” gave positive responses in short-term tests, such as inhibition of specific binding of ^3^H-TPA to a particulate fraction of mouse skin and induction of ornithine decarboxylase in mouse skin, seed oil was thought to contain an active phorbol ester, which binds to a phorbol ester receptor and induces cell proliferation. A two-stage chemical carcinogenesis experiment on mouse skin initiated with DMBA, followed by repeated applications of “irritant fraction I”, produced 36% tumor-bearing mice in week 30. The results clearly showed that “irritant fraction” of seed oil contains phorbol esters with tumor-promoting activity. This preliminary experiment strongly encouraged us next to purify an active phorbol ester from seed oil (Horiuchi et al. 1987).

**Fig. 3:**
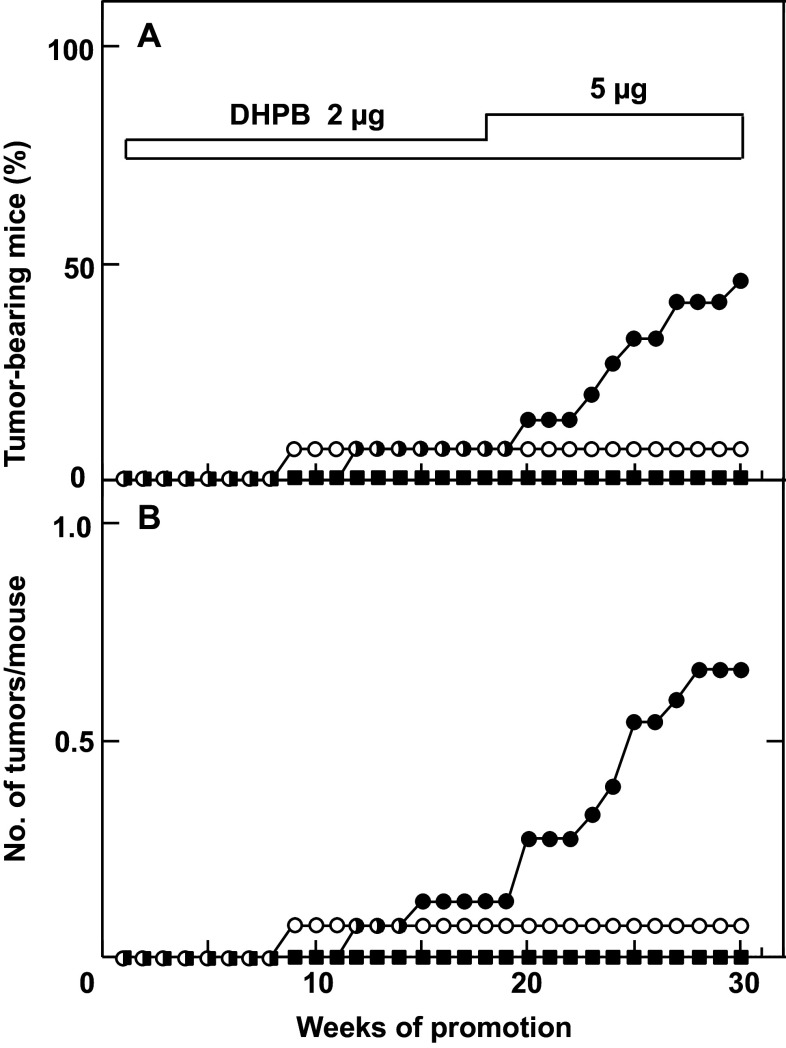
Tumor-promoting activity of DHPB on mouse skin initiated with DMBA. **a** Percentage of tumor-bearing mice, groups treated with DMBA alone (*filled square*), DHPB alone (*circle*), and DMBA plus DHPB (*filled circle*), and **b** number of tumors per mouse, groups treated with DMBA alone (*filled square*), DHPB alone (*circle*), and DMBA plus DHPB (*filled circle*)

Using numerous steps of purification, Mitsuru Hirota and his associates obtained 3 mg of one major compound (Compound I) from 4.6 L seed oil and 19 mg Compound I from 9.8 kg seeds of *Jatropha curcas* (Hirota et al. 1988). Based on the results of spectroscopic analyses of Compound I and its chemical degradation products, its structure was proposed as an intramolecular 13,16-diester of 12-deoxy-16-hydroxyphorbol, i.e., 12-deoxy-16-hydroxyphorbol-4′-[12′,14′-butadienyl]-6′-[16′,18′,20′-nonatrienyl]-bicyclo[3.1.0]hexane-(13-*O*)-2′-[carboxylate]-(16-*O*)-3′-[8′-butenoic-10′] ate (abbreviated to DHPB, Fig. 1) (Hirota et al. 1988). Two-stage chemical carcinogenesis experiments were conducted with 2 μg of DHPB, since topical applications of 10 μg DHPB twice a week on mouse skin killed 25% of the tested mice within 1 week. One week after initiation with 100 μg DMBA, 2 μg of DHPB were applied topically on mouse skin twice a week until week 17. The first tumor appeared at week 12. Because the tumor incidence of the group did not increase as expected, the amount of DHPB was increased from 2 to 5 μg per application from week 18. The percentage of tumor-bearing mice increased gradually from week 20 and reached 46.7% in week 30, and the average number of tumors per mouse in the group treated with DMBA plus DHPB was 0.6 (Figs. 1, 3). All the tumors seemed to be papillomas, not carcinomas. The group treated with DMBA alone yielded only one tumor among 15 mice, and the group treated with DHPB alone did not produce any tumors. These results precisely demonstrated that DHPB represents a new type of phorbol ester class associated with tumor-promoting activity. It is important to note that the tumor-promoting activity of DHPB was slightly weaker than that of TPA, based on biochemical and biological activities (Fig. 1). The weaker activity of DHPB might be explained by the structural difference between DHPB and TPA as follows: (1) the alcohol moiety, i.e., 12-deoxy-16-hydroxyphorbol of DHPB and phorbol of TPA; (2) the acid moieties, i.e., the unsaturated acid of DHPB and saturated acids of TPA; and (3) the conformation of DHPB seeming more flexible than that of TPA. Thus, this flexibility might account for the weak tumor-promoting activity of DHPB. From the results of purification, we estimated that 1 ml of seed oil contains 1 μg DHPB, which is also a major irritant in seed oil. Therefore, DHPB is thought to be a major tumor promoter found in seed oil of *Jatropha curcas* (Hirota et al. 1988). The significance of this investigation alerted Thai scientists and Thai people to the danger of direct contact with seed oil, and it introduced the importance of primary cancer prevention (Suttajit et al. 1990).

In 2002, Martin Mittelbach and his associates isolated six unstable intramolecular diterpene esters from seed oil, and elucidated their chemical structures. ^1^H NMR and ^1^H-^1^H COSY NMR data of compound 2 were almost identical to our previously reported DHPB, except for small differences of the chemical shift values, which can be explained by the use of CD_2_Cl_2_, instead of CDCl_3_ as solvent (decomposition of the compounds was reduced using CD_2_Cl_2_) (Fig. 1, named *Jatropha factor* C_1_) (Haas et al. 2002). The dicarboxylic acid moieties of *Jatropha factor* C_1−5_ contain a bicycle[3.1.0]hexane unit, and *Jatropha factor* C_6−7_ contain a cyclobutane unit, which was found for the first time within this class of compounds. Five of these phorbol esters are newly discovered natural products (Haas et al. 2002). A high-speed countercurrent chromatography identified more than 15 phorbol esters according to the same quasi-molecular ion peak, a very similar sequence-specific fragment ion along with UV absorption spectrum (Hua et al. 2015). However, tumor-promoting activity was not determined in two-stage chemical carcinogenesis experiments on mouse skin.

Even though seed oil was found to contain the tumor promoter DHPB and other phorbol esters, seed oil still attracted attention as the source of an efficient and feasible biomass-based fuel, and oil extracted seed cake confirmed as livestock feed and fertilizer. However, oil cake and seed cake of *Jatropha curcas* did indeed contain residual toxic phorbol esters: the yield data for mature plantations suggest 4 and 5 tonnes of seed per hectare, equivalent to 1.5 tonnes of oil per hectare (Matsuno et al. 1984; Foidl et al. 1996).

## Toxicity of phorbol ester in seed oil and seed cake determined by various bioassays

### Phorbol esters

The extract of seeds showed molluscicidal activity against two species of snails, *Oncomelania hupensis* and *Biomphalaria glabrata*: 4β-Phorbol-13-decanoate killed both snail species at a concentration of 0.001% (10 ppm) (Liu et al. 1997). The snail bioassay was the most sensitive, with 100% mortality at 1 μg of phorbol esters. Thus, snail bioassay could be used to screen the toxic phorbol ester in various products (Devappa et al. 2012). Phorbol esters were given by intragastric administration to Swiss Hauschka mice, and the LD_50_ for male mice was 27.34 mg/kg body mass. Histopathological studies on the organs of the dead mice revealed the followings: the lowest dose (21.26 mg/kg body mass) did not show any significant abnormal changes in the organs; the doses >or 32.40 mg/kg body mass showed diffuse haemorrhage in the lungs, glomerular sclerosis, and atrophy in the kidneys; the highest dose of 36.00 mg/kg body mass induced multiple abruption of cardiac muscle fibers and anachromasis of cortical neurons. These results would help in developing safety measures for pharmaceutical and agricultural application of seed oil (Li et al. 2010). The toxicity of the phorbol esters fractions was estimated by bioassays with three aquatic species of snail (*Physa fontinalis*), brine shrimp (*Artemeia salina*), and daphnia (*Daphnia magna*), along with microorganisms. EC_50_ at 48 h was 0.33 ppm for snail, 26.48 ppm for artemia, and 0.95 ppm for daphnia. The minimum inhibitory concentration was 215 ppm for *Streptococcus pyogenes* and *Proteus mirabilis*, 251 ppm for *Pseudomonas putida*, 58 ppm for *Fusarium* species, and 70 ppm for *Curvularia lunata* (Devappa et al. 2012). It is well known that one of the most potent tumor promoters, TPA, is a strong skin irritant, which histopathologically induces redness, inflammation, and oedema. Similarly, topical applications of phorbol ester-rich extract to the models of reconstituted human epithelium and human corneal epithelium showed marked oedema, presence of less viable cell layers, necrosis and/or partial tissue disintegration in epithelium, and increased inflammatory factors, including interleukin-1α and prostaglandin E_2_. Researchers thus advised handlers to use protective gloves and glasses when handling products of *Jatropha curcas* (Devappa et al. 2013). Using TPA as a model compound, seed oil was directly applied to Madin-Darby canine kidney (MDCK) cells, and activation of protein kinase C and expression of cyclooxygenase-2 (COX-2) were measured. The response of MDCK cells to seed oil was expressed in terms of TPA toxic equivalent (TEQ), which provides a quantitative method to report the inflammatory potential of phorbol ester in seed oil (Pelletier et al. 2015). In addition, varieties of *Jatropha curcas* lacking phorbol ester already exist in Mexico, and non-toxic (edible) varieties could be developed by disrupting genes in the phorbol ester biosynthetic pathway (King et al. 2009).

### Seed and kernel cakes

Seed’s cakes contain a variety of toxins. Even though phorbol ester was not detected in cakes by high-performance liquid chromatography, the cakes inhibited plant seed germination and root growth, and were highly toxic to carp fingerlings. Seed cake exerted acute toxicity on zebrafish embryos, and the concentration of 2.15 g/l resulted in 100% mortality. The observation indicated that phorbol ester remained in seed cake even after extraction (Hallare et al. 2014).

## Degradation of phorbol ester for utilization of seed oil and seed cakes

It is important to study the degradation method of phorbol ester to reduce the toxicity in seed oil and seed cake, the latter of which is generated as a by-product after extraction of seed oil. However, seed cake still containing toxic phorbol ester is used as a fertilizer. Since seed oil and seed cakes are sources of eco-toxicological concern, numerous researchers have studied the degradation method of phorbol ester. Phorbol esters from seed cake were degraded after 21 days at 130 g/kg moisture at room temperature. The rate of degradation was stimulated by increases in temperature and moisture (Devappa et al. 2010). Heat treatment at either 120 °C or 220 °C for 1 h followed by mixing with 10% adsorbing bentonite and nanoparticles of zinc oxide (100 μg/g) plus 4% NaHCO_3_, followed by a 4-week incubation yielded the final product containing almost no phorbol ester (0.04–0.05 mg/g). Cytotoxity tests using mouse fibroblast L929 and normal human dermal fibroblast cell lines confirmed the complete elimination of phorbol ester (Sadubthummarak et al. 2013). Defatted seed cakes contain a high percentage of crude protein, relatively little acid detergent fiber and neutral detergent fiber, while the methanolic extract of seed cakes contains gallic acid, pyrogallol, rutin, myricetin, daidzein, and saponins. The amount of phorbol ester in the methanolic extract was 3.1 ± 0.1 mg/g dried materials by HPLC analysis. The methanolic extract exhibited antioxidant activity that was comparable to β-carotene (Oskoueian et al. 2011). The presence of toxic phorbol ester thus set a limit to utilization of seed cake as livestock feed. *Pseudomonas aeruginosa* PseA strain completely degradated phorbol ester under solid-state fermentation at 30 °C, pH 7.0, and relative humidity 65% (Joshi et al. 2011). The fermentation of seed cake with *Streptomyces fimicarius* YUCM degraded total toxicity by more than 97% in 9 days, and became non-toxic to both plants and carp fingerlings; it also significantly promoted tobacco plant growth. The fermentation made it possible to transform the toxic seed cake to bio-safe animal feed or organic fertilizer, thus removing environmental concerns (Wang et al. 2013). When seed cake was fermented with a fungus species, *Cunninghamella echinulata* CJS-90, the maximum degradation of phorbol ester was 75% (Sharath et al. 2014). In addition, non-pathogenic fungi, such as *Trichoderma harzianum, Paecilomyces sinensis, Cladosporium cladosporioides*, and *Fusarium chlamydosporum*, significantly degraded phorbol ester, with loss of over 92.2–99.7% in broth culture, suggesting that the fungi are potential microbes for the detoxification of phorbol ester (Najjar et al. 2014). Cihera rice bran lipase also showed enzymatic degradation of phorbol ester in seed cake (Hidayat et al. 2014). Cultivation of *Jatropha curcas* in farming may cause leaching of phorbol ester into soil. Seed oil was incubated with clay or black soil under sunlight for different periods of time: under sunlight, phorbol ester decreased to a non-detectable level within 6 days, whereas the level and toxicity of phorbol ester remained little changed when the seed oil was incubated in darkness (Yunping et al. 2012). Although phorbol ester is easily degraded by various methods, resulting seed cake should be carefully tested for toxicity: the seed contains toxic compounds, such as phorbol ester and curcin, the latter of which is a type-1 ribosome inactivating protein (He et al. 2011). One publication about DHPB reported the degradation of active DHPB by esterase KM109 from soil bacterium into non-toxic compound, with reduced cell transforming activity (Nakao et al. 2015). Recently, a new method was developed for the complete utilization of seeds: briquettes of seed cake were made using a vertical extruding machine, and producer gas was obtained by gasifying the briquettes in a downdraft gasifier. Both producer gas and biodiesel from seed oil were used to generate electricity, so the two ways indicate the complete utilization of seed and seed cake without causing any environmental pollution (Raheman and Padhee 2016).

## New pharmaceutical compound from seed of *Jatropha curcas*

**Fig. 4:**
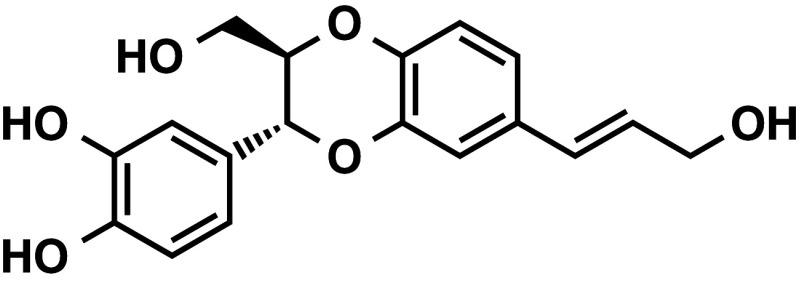
Structure of isoamericanol A isolated from *Jatropha curcas*. Isoamericanol A is not a phorbol ester, and inhibits growth of human cancer cell lines

*Jatropha curcas* was used as a traditional folk medicine for antiviral, analgesic, and/or antidotal efficacies in Thailand, India, other Asian and African countries, and also South America. The genus name *Jatropha* is derived from the Greek *iatrós* (doctor) and trophé (food) which implies medicinal use (Debnath and Biesen 2008). Masaaki Tokuda and his associates at Kagawa University isolated a neo-lignan isoamericanol A, which is not a phorbol ester, from seed extract of *Jatropha curcas* (Fig. 4) (Katagi et al. 2016). Isoamericanol A was previously isolated from seed of *Phytolacca americano* L. and reported to enhance choline acetyltransferase in a cultured neuronal cell system (Fukuyama et al. 1992). Tokuda’s research group reported that isoamericanol A inhibits growth of human cancer cell lines, such as breast cancer (MCF-7 and MDA-MB231), liver cancer (Huh-7), and cervical cancer (HeLa) within a dose range of 25–100 μg/ml dose-dependently. Flow cytometry analysis showed that isoamericanol A induces cell cycle arrest at G_2_/M for MCF-7 cells, and TUNEL staining showed that a concentration of isoamericanol A 50 μg/ml induced apoptotic cells by 24% after 72 h. The treatment of MCF-7 cells with isoamericanol A enhanced expression of cell cycle-related genes, such as *GADD45A, BTG2, p21*, and *FAS*, and it reduced expression of *cyclin B*_*1*_, *cyclin B*_*2*,_ and *CDK1* genes (Katagi et al. 2016). Whether isoamericanol A inhibits carcinogenesis *in vivo* and how toxic it is for humans remains to be further investigated. It is of importance to note that isoamericanol A opened a molecular pharmacological field with *Jatropha curcas*: the long tradition of folk medicine indicates that the seed oil and seed cake of *Jatropha curcas* are not only biofuel and fertilizer, but also a source of pharmaceutical modern research (Debnath and Bisen 2008).

## Tumor promotion and progression with endogeneous tumor promoters in human carcinogenesis

A tumor promoter stimulates clonal growth of the initiated cells and theoretically produces benign tumors with the least cancer in rodents. Tumor promotion by phorbol esters, such as TPA or DHPB, has only been studied on mouse skin, which involved protein kinase C activation (Fujiki et al. 1984a, 1991). Considering the mechanism of tumor promotion in various organs, we think that there must be a general mechanism of tumor promotion in most cancer-affected organs, so we were fortunate to discover okadaic acid as a new tumor promoter on mouse skin. Okadaic acid showed tumor-promoting activity similar to TPA, and it is a potent inhibitor of protein phosphatases 1 and 2A (PP1 and PP2A). The results indicated that okadaic acid induces tumor promotion through a mechanism of action different from TPA and DHPB (Fujiki and Suganuma 1993). Therefore, it was exciting to find that okadaic acid induced tumor promotion in male SD rat glandular stomach initiated with *N*-methyl-*N’*- nitro-*N*-nitrosoguanidine (MNNG) (Suganuma et al. 1992). Moreover, the i.p. administrations of other potent inhibitors of PP1 and PP2A, such as microcystin-LR, and nodularin, into mice induced tumor promotion in rat liver initiated with diethylnitrosamine (DEN) (Nishiwaki-Matsushima et al. 1992; Ohta et al. 1994). All the results strongly indicated that inhibition of PP1 and PP2A is a general mechanism of tumor promotion in various organs of rodents, thus revising the historical concept of tumor promotion (Fujiki and Suganuma 1993, 2011).

Activation of protein kinase C by TPA and DHPB induces transient accumulation of phosphoproteins, resulting in enhancement of gene expression, while inhibition of PP1 and PP2A by okadaic acid class tumor promoters sustains phosphorylation of proteins, resulting in enhancement of gene expression. Among various gene expressions induced by both types of tumor promoter, we think that there is an essential gene commonly involved in signal transduction for tumor promotion in various organs: based on evidence that human fibroblasts treated with okadaic acid and TNF-α showed biochemical similarity, okadaic acid was thought to mimick the phorphorylation pattern induced by TNF-α or IL-1 (Guy et al. 1992). We were fortunate to find that both TPA and okadaic acid induced *TNF-α* gene expression in mouse skin and that okadaic acid, microcystin-LR, and nodularin induced *TNF-α* gene expression in various target organs, including mouse skin, rat glandular stomach, and rat liver (Fujiki and Suganuma 1993, 2011). In addition, treatments of BALB/3T3 cells with okadaic acid and with human TNF-α both stimulated transformation of BALB/3T3 cells initiated with 3-methylcholanthrene (MCA), indicating that okadaic acid and TNF-α have cell transforming activity in cell culture (Komori et al. 1993). Moreover, the tumor-promoting activity of TNF-α was confirmed by two-stage carcinogenesis experiments on the skin of TNF-α deficient (TNF-α^−/−^) 129/Svj mice and TNF-α^+/+^ CD-1 mice treated with DMBA plus okadaic acid and with DMBA plus TPA (Suganuma et al. 1999). Because TNF-α is induced as a proinflammatory cytokine and activates NF-κB at molecular level in cells, all the proinflammatory cytokines and chemokines are now understood to be endogenous tumor promoters in human carcinogenesis (Fujiki et al. 2013). Our study made it possible to increase tumor promotion possibilities from chemical tumor promoters to endogenous tumor promoters.

When we published a review article entitled “Tumor promotion by inhibitors of protein phosphatases 1 and 2A: the okadaic acid class of compounds” (Fujiki and Suganuma 1993), we raised three hypotheses concerning how the okadaic acid pathway is related to human cancer: the first hypothesis was simply the exposure of the okadaic acid class compounds, the second was the involvement of endogenous protein inhibitors of PP1 and PP2A, and the third was that the effects of okadaic acid mimicked those of TNF-α and IL-1 in cells, as presented above. Concerning the second possibility, endogenous protein inhibitors of PP1, named inhibitor-1 (18 kDa) and inhibitor-2 (22 kDa), were previously found in cells (Cohen 1989). Recently, a chimeric *set-can* transcript was detected in a bone-marrow sample from a patient with acute undifferentiated leukemia, and its DNA sequence predicted an SET-CAN protein of 155 kDa in the cells (von Lindern et al. 1992). Moreover, SET and CIP2A were found to be endogenous inhibitors of PP2A, and SET, CIP2A, and pS62-MYC proteins are commonly overexpressed in human breast cancers (Sablina et al. 2010; Janghorban et al. 2014). Now, tumor promotion in human cancer is understood as a disease that affects humans in two ways: the up-regulation of proinflammatory cytokines and chemokines and the inhibition of PP2A by PP2A inhibitors (Fujiki and Suganuma 1993; Fujiki et al. 2013). Thus, our two hypotheses of okadaic acid pathways were proved to be involved in human cancer development, and all the results indicate that humans are always at risk of tumor promotion. With these physiological conditions, we need to establish our own cancer prevention strategy, to reduce TNF-α and IL-1 and inactivate NF-κB. Considering this theory, primary cancer prevention should include not only avoiding contact with tumor promoter and carcinogen in our environment, but also avoiding inflammation in our body.

## Concept of primary and tertiary cancer preventions

**Fig. 5:**
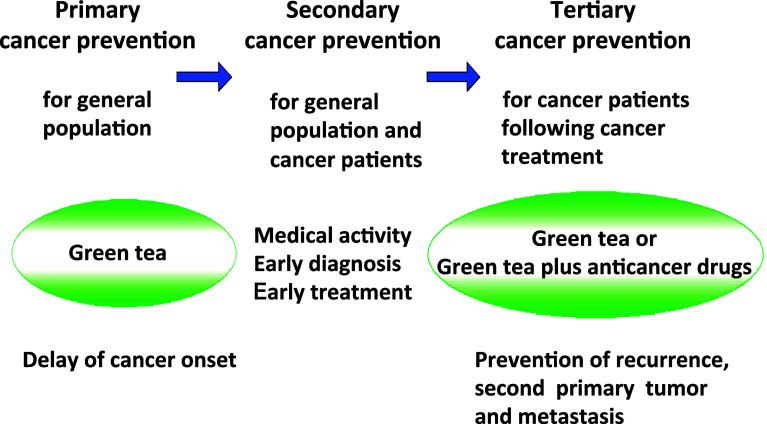
Primary, secondary, and tertiary cancer preventions in humans. Primary cancer prevention is for general population, resulting in reduced cancer incidence, secondary cancer prevention means early cancer diagnosis and treatment for general population and cancer patients at the clinics, and tertiary cancer prevention is for cancer patients following cancer treatment

There are primary, secondary, and tertiary cancer preventions in the clinics (Fig. 5). Since we are exposed to numerous tumor promoters, mutagens, and carcinogens in our environment, we should avoid direct contact with such compounds from the standpoint of primary cancer prevention. When we identified tumor-promoting activity of a new phorbol ester, DHPB, in seed oil of *Jatrapha curcas* in 1988 (Hirota et al. 1988), awareness of the hazard and caution in public use of seed oil in Saboodam was strongly publicized in Thailand and the other Asian countries (Suttajit et al. 1990; Fujiki et al. 1987, 1991). Moreover, non-smoking is a typical example of primary cancer prevention: it means that primary cancer prevention is an action of self-prevention in every-day life. In addition, Michael B. Sporn at National Cancer Institute, USA, coined the term “Cancer chemoprevention”, and defined it as “prevention of the occurrence of cancer by administration of one or several compounds” (Sporn et al. 1976). The clinical prevention trial of primary breast cancer was conducted by administration of tamoxifen for 1 year to senior women and a high-risk group from 35 to 59 years. Tamoxifen significantly prevented 50% of primary breast cancer development (Fisher et al. 1998). Furthermore, Kei Nakachi and Kazue Imai found that cancer onset in female patients who consumed over 10 Japanese-size cups (120 ml/cup) of green tea per day was 7.3 years later than that of female patients who had consumed less than three cups per day. It is important to note that the delay of cancer onset is a significant biomarker for primary cancer prevention in humans (Nakachi et al. 2000). Now, primary cancer prevention is widely practiced in the world (Fig. 5).

The process from tumor promotion to progression in human carcinogenesis gains significance in multistage carcinogenesis. The multistage carcinogenesis theory developed by Bert Vogelstein showed that the first tumor of the colorectum appears as an adenoma and becomes a big adenoma; genetic changes then accumulate in the cells and become malignant tumor (Vogelstein et al. 1988). It takes about 20–30 years from the first genetic change in the cells to clinical appearance of cancer and metastasis in patients. In such cases in hospital, endoscopic polypectomy is conducted to prevent the growth of adenomas. This activity is an example of secondary cancer prevention (Fig. 5). Patients often have recurrence of adenoma several years after polypectomy: Hisataka Moriwaki’s group at Gifu University, in collaboration with us, conducted double-blind randomized clinical phase II prevention trial for recurrence of colorectal adenomas, and found that drinking ten Japanese-size cups of green tea, supplemented with green tea tables, 51.6%, significantly prevented recurrence of colorectal adenomas in patients (Shimizu et al. 2008). This prevention is vital evidence of tertiary cancer prevention (Fig. 5) (Fujiki et al. 2012).

## Summary

The discovery of a new tumor promoter, DHPB in seed oil of *Jatropha curcas*, was almost 30 years ago. However, even though *Jatropha curcas* contains toxic phorbol ester, the seed oil attracted great attention as a renewable oil or biodiesel, and the extracted seed and kernel cakes were use as fertilizer and livestock feed in the world. Due to the wide utilization value of seed oil and seed cake, numerous investigators expanded the science of *Jatropha curcas* to toxicological assay, degradation of phorbol ester, and a new pharmaceutical compound based on traditional folk medicine. Since phorbol ester is a tumor promoter on mouse skin, tumor promotion in human cancer was studied using inflammatory proteins, such as cytokines and chemokines, and endogenous inhibitors of PP1 and PP2A. Finally, the significance of cancer prevention was acknowledged.
